# The effects of motor adaptation on ankle isokinetic assessments in older drivers

**DOI:** 10.6061/clinics/2018/e303

**Published:** 2018-07-23

**Authors:** Angelica Castilho Alonso, Guilherme Carlos Brech, Rita de Cássia Ernandes, Douglas Rodrigues, Sérgio Ayama, Alexandra Carolina Canonica, Natália Mariana Silva Luna, Sileno da Silva Santos, Luis Mochizuki, Mark Peterson, Luiz Eugênio Garcez-Leme, Júlia Maria D’Andréa Greve

**Affiliations:** I Laboratorio de Estudos do Movimento, Instituto de Ortopedia e Traumatologia, Hospital das Clínicas HCFMUSP, Faculdade de Medicina, Universidade de Sao Paulo, SP, BR; II Departamento de Ciěncias do Envelhecimento da Universidade São Judas Tadeu (USJT), São Paulo, SP, BR; III Escola de Artes, Ciěncias e Humanidades, Universidade de São Paulo (USP), São Paulo, SP, BR; IV Department of Physical Medicine and Rehabilitation, University of Michigan-Medicine, Ann Arbor, Michigan, United States; V Grupo de Ortogeriatria, Instituto de Ortopedia e Traumatologia, Hospital das Clínicas HCFMUSP, Faculdade de Medicina, Universidade de Sao Paulo, SP, BR

**Keywords:** Ankle, Muscle Strength, Muscle Strength Dynamometer, Older Drivers

## Abstract

**OBJECTIVES:**

This study sought to analyze the extent of motor adaptation in ankle plantar flexors and dorsiflexors among older drivers during clinical isokinetic testing.

**METHODS:**

One hundred older adults (70.4±5.7 years) participated in two bilateral ankle plantar flexor and dorsiflexor isokinetic assessments at 30°/sec. Peak torque (PTQ), PTQ adjusted for body weight (PTQ/BW), and total work (TW) were analyzed.

**RESULTS:**

On the dominant side, PTQ/BW and TW were significantly greater for the second plantar flexion test than were those for the first such test (*p*<0.001), whereas PTQ, PTQ/BW, and TW (*p*<0.001) were significantly greater for the second dorsiflexion test than were those for the first such test. On the non-dominant side, plantar flexion PTQ and TW were significantly lower for the second test than were those for the first test (*p*<0.001).

**CONCLUSION:**

Older drivers demonstrated better performance with the dominant limb on the second test. The low variability in test execution showed the existence of a motor adaptation effect for the tested movements, despite the short recovery period between the assessments.

## INTRODUCTION

The ability to execute precise and rapid braking is an important determinant of safe driving. Vision, cognition, mobility and strength are important attributes involved in complex coordination during driving-related braking 1,2. Compared with other drivers, older adults have significantly lower ankle plantar flexor strength, a characteristic associated with worse braking time performance 3.

Measurements of muscle strength are a feature of all studies related to motor evaluations of drivers. Such evaluation is part of the Assessment of Driving Related Skills (ADReS) 4.

Isokinetic dynamometry provides an objective measure of muscular functional parameters. Motor adaptation is characterized by acute improvements in physical function and muscle strength capacity 5-7. An issue related to motor learning or adaptation is the extent to which the effects of motor adaptation can modify the results of objective assessments, even during the short period between repeated isokinetic tests 7,8.

However, it is unclear whether acute motor adaptation extends to all muscle groups and populations. Thus, the aim of this study was to analyze the effects of acute motor adaptation for older drivers who were performing two isokinetic tests of the ankle joint that involved plantar flexor and dorsiflexor movements.

## METHODS

### Experimental design and subjects

This study was performed at the Institute of Orthopedics and Traumatology, Clinics Hospital, School of Medicine, University of São Paulo, with approval from the Ethics Committee of the University of São Paulo (number 0468/10).

One hundred older patients (>65 years; 50 men and 50 women) from the geriatric ambulatory department of Clinics Hospital of the University of São Paulo School of Medicine were evaluated.

The inclusion criteria for both groups were preserved cognitive aspects, as evaluated using the Mini-Mental State Examination (MMSE); not being on medication that could interfere with driving ability; no history of lower limb injury in the preceding six months; no prior surgery that could have altered mobility of the lower limbs; no restrictions on ankle, knee, or hip mobility; and a normal clinical gait without claudication. The exclusion criterion was an inability to perform the muscle strength test or undergo a physical examination.

### Isokinetic evaluation

The maximum dynamic concentric strengths of plantar flexion and dorsiflexion for the dominant and non-dominant ankles were measured using a Biodex^®^ Multi-joint System 3 (Biodex Medical^TM^, Shirley, NY, USA). Subjects were placed using the same methodology that our group employed in a prior study 3. Three submaximal attempts were performed to allow for familiarization with the equipment. Subsequently, two sets of five maximal dynamic repetitions were conducted at an angular velocity of 30°/s, with a one-minute interval between these sets. During each test, constant standardized verbal encouragement was provided to maximize the subject's effort during contractions 7.

### Statistical analyses

Descriptive analyses of the sample are presented as the means, standard deviations, minimum values, and maximum values. The Kolmogorov–Smirnov test was applied to ensure that the data followed a Gaussian distribution. Two-way Anova with repeated measures was used to evaluate the effect of motor adaptation between the first and second trials and the effect of lower limb dominance with respect to muscle strength. The threshold for significance was set as *p*<0.05. The Statistical Package for the Social Sciences (SPSS) (v.20) for Windows was used to perform all the statistical analyses.

## RESULTS

The subjects’ sociodemographic characteristics are shown in Table 1.

Means and standard deviations of examined variables, including peak torque (PTQ), PTQ adjusted for body weight (PTQ/BW), total work (TW), and agonist/antagonist ratio for plantar flexion and dorsiflexion, are shown in Table 2. Muscle strength was significantly higher in the dominant limb than in the non-dominant limb. On the dominant side, plantar flexors produced significantly higher values for PTQ/BW and TW for the second test than for the first test, whereas PTQ, PTQ/BW, and TW for dorsiflexion were significantly higher for the second test than for the first test. For the non-dominant lower limb, plantar flexors exhibited significantly lower values for PTQ and TW during the second test than during the first test. There were no significant differences in dorsiflexion outcomes for the non-dominant limb. The agonist/antagonist ratio decreased significantly for both lower limbs from the first assessment to the second assessment.

The means and standard deviations for coefficients of variation (CVs), minimums and maximums are shown in Table 3. CVs were significantly lower for the second test than for the first test for plantar flexion on the dominant side (*p*<0.001). CVs were significantly higher for the dominant limb than for the non-dominant limb.

## DISCUSSION

The primary findings of this research were that multiple repetitions of the tests led to neuromuscular integration of motor learning, which resulted in more reliable outcomes. The relative discomfort (restraint) caused by equipment positioning can inhibit assessments of individuals, affecting their performances during the execution of tests; therefore, the effect of adaptation is highly relevant to this study.

For the dominant ankle, better results were obtained for the second test than for the first test for the PT/BW and TW variables for plantar flexion muscle strength; this finding is consistent with those reported in other studies 7,8. This phenomenon suggests an adaptation and learning effect with respect to the use of computerized strength testing equipment. Driving ability is a complex task that demands various skills and physical capacities, such as muscle strength, which is one of the most important predictors of driving safety 3,9. In a previous study, ankle plantar flexor muscles were evaluated because of the specificity of movements associated with driving, and a robust relationship between these muscles and the dynamic task of braking was observed. The results showed that lower muscle strength during plantar flexion was associated with worse braking time performance 3. Therefore, older drivers took longer to brake a car, which could cause accidents in certain situations 1.

In another study involving knee flexion and extension assessment, better performance was observed on a second trial than on a first trial completed during the same session 7; this observation is consistent with our findings for the ankle joint.

For the non-dominant ankle, our assessments revealed lower strength for the second trial than for the first trial, with only plantar flexor PTQ and TW exhibiting improvement for the second trial. Certain psychological factors can explain this phenomenon. Dominance itself leads to higher cortical representation of muscle on the dominant side; as a result, motor adaptation is faster on that side based on the recruitment of more motor units during movement 10. On the non-dominant side, only plantar flexor performance was better for the second trial than for the first trial. Higher strength and activity for plantar flexors than for other muscles during daily activities (gait, balance and orthostatic activities) could potentially explain this finding. Driving a vehicle with a mechanical transmission involves the use of the left ankle during top gear, which could make learning easier for muscles associated with this ankle. A study that compares performance between older drivers and non-drivers might shed light on this possibility 11.

The dominant limb had better performance for all isokinetic variables for both tested muscle groups. In their meta-analysis, Mcgrath et al. 10 affirm that there are significant differences between the performances of thigh muscles for the dominant and non-dominant leg in adults; specifically, functional asymmetries have been observed for the lower legs of athletes with different skills and specializations 10,12. However, results have suggested that vehicle direction (in particular, the force designed to press the accelerator and brake pedal for approximately 432.8 (±9.2) years) appeared to promote a selective muscle pattern, and may generate imbalances between the dominant and non-dominant limbs.

The CVs of repetitions decreased for the second trial only for the dominant plantar flexors, which reflects these muscles' better capacity for contraction maintenance during subsequent repetitions. Lower CVs indicate greater muscle fatigue resistance and the higher capacity of a muscle to recruit motor units. In combination, these capabilities allow for constant torque production during all repetitions 6,13,14.

The agonist/antagonist ratio for the dominant and non-dominant limbs decreased for the second trial. This outcome might indicate a decrease in dorsiflexor concentric PTQ or an increase in plantar flexor PTQ that resulted in an imbalance in the tested joint 12,15. These results confirm the importance of task specificity for driving assessments and suggest that driving creates imbalances between muscle group agonists/antagonists for the same limb.

One of the most important clinical implications of performing muscle strength assessment for healthy elderly individuals by utilizing two tests in a short time is to use the second test to improve the trustworthiness of the results.

Our results demonstrate that relative to an initial test, on a second test of plantar flexion and dorsiflexion, older drivers exhibit better performance with their dominant limb and lower test execution variability, suggesting that they adapted to the execution of the proposed movements for the second test, even with a short recovery period between tests.

## AUTHOR CONTRIBUTIONS

Alonso AC was responsible for the data collection, manuscript writing and data analysis. Ernandes RC was responsible for the data collection and manuscript revision. Wenzel DR, Ayama S and Canonica AC were responsible for the data collection and manuscript writing. Luna NM and Santos SS were responsible for the manuscript writing. Brech GC was responsible for research design and manuscript revision. Mochizuki L was responsible for the data analysis and interpretation. Peterson M was responsible for the manuscript language and grammar revisions. Garcez-Leme LE and Greve JM were responsible for the research supervision and manuscript revision.

## Figures and Tables

**Table 1:** Demographic characteristics and self-reported information about education and driving.

Older drivers		
M (SD)		
Age (years)	70.4 (5.8)	
Years licensed	42.8 (9.2)	
Years of education	12.6 (3.0)	
Body mass (kg)	64.2 (9.0)	
Height (cm)	1.60 (0.6)	
BMI (kg/m^2^)	25.06 (3.7)	
**Physical activity (IPAQ) %**		
Irregular activity A	64	
Irregular activity B	36	
**Dominant lower limb %**		
Right	100	
Left	0	
**Occupation %**		
Retired	93	
Salaried	7	
**Family income %**		
>½ to 1 MW	5	
>1 to 2 MW	07	
>2 to 3 MW	08	
>3 to 5 MW	37	
>5 to 10 MW	21	
>10 to 20 MW	10	
>20 MW	03	
Refused to answer	9	

M: mean; SD: standard deviation; IPAQ: International Physical Activity Questionnaire; BMI: body mass index; MW: minimum wage.

Note: Irregular activity A: individuals who satisfy at least one of the recommended criteria for activity frequency or duration; Irregular activity B: individuals who do not satisfy any of the recommended criteria for activity frequency or duration.

**Table 2:** Comparisons of isokinetic data (for the variables PTQ, PT/BW, TW and Agon/antag ratio) for the first and second tests of the dominant and non-dominant limbs.

Variables	PTQ (Nm)		PT/BW (Nm/kg)		TW (J)		Agon/antag ratio (%)	
	First test M (SD)	Second test M (SD)	First test M (SD)	Second test M (SD)	First test M (SD)	Second test M (SD)	First test M (SD)	Second test M (SD)
**Plantar flexors**								
Dominant	46.1 (26.4)	51.6 (21.9)	63.0 (27.7)	74.2 (30.9)^a^	69.1 (43.7)	83.3 (52.8)^a^	61.3 (28.2)	51.6 (20.8)^a^
Non-dominant	24.6 (10.2)^b^	23.2 (5.8)^a^,^b^	34.7 (11.8)^b^	33.1 (7.6)^b^	43.1 (15.2)^b^	40.7 (13.0)^a^,^b^	60.1 (39.9)	54.7 (38.4)^a^
**Dorsiflexors**								
Dominant	47.4 (22.5)	53.1 (23.3)^a^	68.5 (31.7)	75.5 (32.8)^a^	73.3 (51.8)	83.8 (51.6)^a^		
Non-dominant	24.8 (8.0)^b^	24.0 (6.3)^b^	34.7 (11.8)^b^	33.1 (7.5)^b^	43.3 (16.9)^b^	41.7 (15.0)^b^		

M: mean; SD: standard deviation; PTQ: peak torque; PTQ/BW: peak torque adjusted for body weight; TW: total work; Agon/antag: agonist/antagonist.

a *p*<0.001 for the difference between the first and second tests.

b *p*<0.001 for the difference between the dominant and non-dominant limbs.

**Table 3:** Coefficients of variation. For the dominant limb, the plantar flexors exhibited significantly lower CVs for the second test. The dominant limb exhibited significantly higher CVs than the non-dominant limb.

Coefficients of variation (CV)		
Variables	CV (%)	
	First test M (SD) MIN-MAX	Second test M (SD) MIN-MAX
**Plantar flexors**		
Dominant	15.3 (10.8) (3.5-60)	11.0 (8.3) (2.6-56.4)^a^
Non-dominant	6.9 (8.9) (0.9-72.1)^b^	6.1 (3.0) (1.8-16.1)^b^
**Dorsiflexors**		
Dominant	14.3 (11.4) (2.1-64.4)	10.8 (11.87) (1.4-100.9)
Non-dominant	7.7 (8.9) (1.7-59)^b^	6.1 (3.4) (1.6-20.2)^b^

M: mean; SD: standard deviation; MIN: minimum; MAX: maximum;

a *p*<0.001 for the difference between the first and second tests;

b *p*<0.001 for the difference between the dominant and non-dominant limbs.
